# Multimodal therapy for treatment of equine back pain: a report of 15 cases

**DOI:** 10.29374/2527-2179.bjvm003321

**Published:** 2021-11-22

**Authors:** Ubiratan Pereira de Melo, Cintia Ferreira

**Affiliations:** 1 Veterinarian, DSc. Faculdade Maurício de Nassau (Uninassau). Campus Natal, RN, Brazil.

**Keywords:** dynamic mobilization exercises, ozone, platelet-rich plasma, shoeing, therapeutic ultrasound, exercícios de mobilização dinâmica, ozônio, plasma rico em plaquetas, ferrageamento, ultrassom terapêutico

## Abstract

Back pain and diseases of the spine are considered significant problems in equine sports and veterinary medicine. This article reports a multimodal approach to the treatment of equine back pain using ozonized platelet rich plasma (PRP), dynamic mobilization exercises, and therapeutic shoeing in 15 horses involved in the vaquejada discipline. Fifteen American Quarter Horses of both sexes engaged in vaquejada in the state of Rio Grande do Norte, Brazil, with a mean age of 8.61 ? 1.73 years were examined at a training center for lower performance diagnostics or back pain. A complete clinical examination was performed on all horses at rest to determine the general conformation and alterations in posture, symmetry, and curvature of the spine. The horses were examined while walking and trotting in straight lines and circles to determine the presence of lameness and/or gait asymmetry. Spinal abnormalities on clinical examination were classified on a scale of 0 to 5 based on the following parameters: degree of response to pain from back palpation, hypertonicity of the back muscles, stiffness of the thoracolumbar joint, and physical dysfunction. After physical examination, ultrasound was performed to identify the cause of the thoracolumbar pain. The therapeutic protocol consisted of the intralesional application of ozonized PRP combined with therapeutic ultrasound, dynamic mobilization exercises, and therapeutic shoeing. All treated animals returned to sports activities at a higher level of performance than at the beginning of the treatment. Six months after treatment, telephone contact was made with the owner or trainer to determine if the back pain had relapsed. None of the animals relapsed during this period, and they participated in vaquejada normally.

## Resumo

Lombalgia e doenças da coluna vertebral são considerados problemas significativos nos esportes equestres e na medicina veterinária. Este artigo tem como objetivo relatar uma abordagem multimodal no tratamento da lombalgia em equinos utilizando plasma rico em plaquetas ozonizado, exercícios de mobilização dinâmica e ferrageamento terapêutico em quinze equinos da disciplina vaquejada. Quinze equinos da raça Quarto de Milha, praticantes da modalidade vaquejada no estado do Rio Grande do Norte, Brasil, ambos os sexos, com idade média de 8,61 ? 1,73 anos, foram examinados em centro de treinamento para diagnóstico de baixo desempenho ou lombalgia. Exame clínico completo foi realizado em todos os equinos em repouso para determinar a conformação geral, alterações na postura, bem como simetria e curvatura da coluna vertebral. Os equinos foram examinados enquanto caminhavam e trotavam em linhas retas e círculos para determinar a presença de claudicação e/ou assimetrias de marcha. As anormalidades da coluna vertebral no exame clínico foram classificadas em uma escala de 0 a 5 com base nos parâmetros: grau de resposta da dor à palpação, hipertonia muscular, rigidez da articulação toracolombar e disfunção física. Após exame físico, foi realizada ultrassonografia para identificação da causa da dor toracolombar. O protocolo terapêutico consistiu na aplicação intralesional de PRP ozonizado associado ao uso de ultrassom terapêutico, exercícios de mobilização dinâmica e ferrageamento terapêutico. Todos os animais tratados voltaram às atividades esportivas com nível de desempenho superior ao demonstrado no início do tratamento. Seis meses após o tratamento, foi realizado contato telefônico com o proprietário ou treinador para saber se a lombalgia havia recidivado. Nenhum dos animais teve recidiva durante este período e estavam disputando provas de vaquejadas normalmente.

## Introduction

In the modern era, equestrian sports are growing in interest among the general public, representing an important economic entity. Horses used in high-performance sports competitions always work close to the limit of their physical capacity, resulting in frequent injuries (Figueiredo et al., 2016).

Back pain and diseases of the spine are considered significant problems in both equine sports and veterinary medicine (Fonseca et al., 2006; Melo & Ferreira, 2020; Barreto et al., 2021). However, reports of their incidence are limited to surveys from general practice in private clinics or educational institutions.

The specific causes of back pain include ligamentous lesions, muscle strain, osteoarthritis and ankylosis of the intertransverse and/or lateral intertransverse joints, fractures of the thoracolumbar (TL) and/or lumbo-pelvic complex, vertebral body osteophytes and spondylosis, impingement of the dorsal spinous processes, degenerative intervertebral disc disease, sacroiliac disease, and osteoarthritis of the synovial intervertebral articulations (facet joints) (Alves et al., 2007; Cousty et al., 2010; Mendes et al., 2013).

Equine back pain can also be caused by problems remote from the spine, i.e., pain referred from a non-spine source. Many horses with back pain also have hindlimb lameness, the most common cause being unilateral or bilateral bone spavin (Melo et al., 2008). Poorly fitting saddles have been suggested to be another cause of back pain and asymmetry in the epaxial muscle mass (Dantas et al., 2019).

Diagnosis of equine back pain can be challenging because the condition presents as a syndrome rather than as specific clinical signs. Thus, despite the availability of sophisticated clinical aids, the definitive diagnosis of equine back injuries is most often made by eliminating other conditions (Mayaki et al., 2020; Melo & Ferreira, 2020).

As the field of equine internal medicine continues to grow, the optimal function of the axial portion of the skeleton has become an important clinical issue in athletic horses (Haussler, 2015). Over the years, a multitude of different medical (Fonseca et al., 2006), surgical, physical medicine, nutritional, and equitation approaches have been applied to horses with back problems (Fantini & Palhares, 2011; Melo & Ferreira, 2020). Therapeutic options are diverse and are typically based on an individual practitioner’s clinical bias or postgraduate training in adjunctive therapies that have been extrapolated from human medicine and applied to horses (Haussler, 2015).

This study describes a multimodal approach for the treatment of equine back pain using ozonized platelet-rich plasma (PRP), dynamic mobilization exercises, and therapeutic shoeing in 15 horses engaged in the vaquejada modality.

## Case report

Fifteen American Quarter Horses of both sexes engaged in vaquejada in the state of Rio Grande do Norte, Brazil, with a mean age of 8.61 ? 1.73 years, were examined at a training center for lower performance diagnostics or back pain.

A complete clinical examination was performed on all horses at rest to determine the general conformation and alterations in posture, symmetry, and curvature of the spine. The horses were examined while walking and trotting in straight lines and circles to determine the presence of lameness and/or gait asymmetry. Oral examination of the horses was performed to rule out the influence of dental pain on gait.

The pectoral region, the dorsal scapular and wither regions, and the epaxial and gluteal muscles were carefully evaluated for abnormal muscle development and left-right asymmetries.

The thoracolumbar region was palpated with firm finger pressure along the dorsal thoracolumbar midline by a single examiner (UPM) to avoid variability in the interpretation of the equine response. The avoidance reaction (i.e., sinking) to palpation was used as an indicator of pain and classified as absent, mild, moderate, or severe.

Thoracolumbar flexibility was assessed using spinal manipulation: thoracolumbar flexion, thoracolumbar extension, lumbar-sacral extension, lumbar-sacral flexion, lateroflexion, and rotation to the left and right.

Spinal abnormalities (Tables 1 and 2) found during clinical examination were graded on a scale of 0 to 5 based on the following parameters: degree of pain response to back palpation, back muscle hypertonicity, thoracolumbar joint stiffness, and physical dysfunction, as suggested by Mayaki et al. (2020).

**Table 1:** Classification of equine back pain according to structural and functional abnormalities of the spinal cord at the time of initial clinical examination in 15 American Quarter Horses in the state of Rio Grande do Norte, Brazil.

Parameter	Abnormality score (grade)					
	0 (absence)	1 (mild)	2 (mild moderade)	3 (moderade)	4 (severe)	5 (incapacited)
Pain response	-	2 (13.33%)	4 (26.66%)	5 (33.33%)	4 (26.66%)	-
Muscle hipertonicity	-	-	4 (26.66%)	6(40.00%)	5 (33.33%)	-
Lameness	8	3 (20.00%)	2 (13.33%)	2 (13.33%)	-	-
Thoracolumbar joint stiffness	-	-	6 (40.00%)	9 (60.00%)	-	-
Physical dysfunction	3 (20.00%)	6 (40.00%)	6 (40.00%)	-	-	-

**Table 2:** Grading of equine back pain based on structural and functional spinal abnormalities 60 days after multimodal therapy in 15 American Quarter Horses in the state of Rio Grande do Norte, Brazil.

Parameter	Abnormality score (grade)					
	0 (absence)	1 (mild)	2 (mild moderade)	3 (moderade)	4 (severe)	5 (incapacited)
Pain response	15 (100%)	-	-	-	-	-
Muscle hipertonicity	15 (100%)	-	-	-	-	
Lameness	15 (100%)	-	-	-	-	-
Thoracolumbar joint stiffness	14	1			-	-
Physical dysfunction	3 (20.00%)	6 (40.00%)	6 (40.00%)	-	-	-

After physical examination, ultrasound was performed to identify the cause of thoracolumbar pain. The entire thoracolumbar region was evaluated in each animal. Before performing the ultrasound examination, the entire area to be evaluated was shaved and washed with a neutral detergent. After cleaning, a water-soluble gel was applied to the skin for better contact with the transducer. A 5-7.5MHz multifrequency linear transducer was used following the protocol described by Fonseca et al. (2006) and Melo and Ferreira (2020) to obtain images of thoracolumbar structures in transversal and longitudinal images to the dorsal axial line.

Supraspinous ligament lesions were characterized according to echogenicity and parallelism of the fibers, and lesions of the interspinous space were classified according to increased echogenicity, presence of hyperechoic points in the space, and reduction or loss of the space. Spinous processes were characterized based on the regularity of the dorsal surface. Kissing spines were considered present when continuity of the bone line between two or more spine processes, with loss of the interspinous space, following the protocol described by Fonseca et al. (2006).

The muscles were characterized by the echogenicity of the fibers (hypoechoic zones) and perimysium (hyperechoic lines that separate the fibers). The presence of a hypoechoic or anechoic gap within the muscle fibers was considered a criterion for the diagnosis of myositis. In chronic cases, a fibrotic area can appear as a circumscribed hyperechoic lesion.

The main ultrasonographic findings are summarized in Table 3. After localization and characterization of the lesions through clinical and ultrasound examinations, clinical treatment was performed.

**Table 3:** Ultrasonographic findings on initial clinical examination of 15 American Quarter Horses in the state of Rio Grande do Norte, Brazil.

Case	Ultrasonographic findings			
	Epaxial myositis	Supraspinous lesion	Multifidus muscle atrophy	Kissing spines
1	Present	Absent	Present	Absent
2	Present	Present	Absent	Absent
3	Present	Present	Present	Present
4	Present	Absent	Present	Absent
5	Present	Present	Present	Absent
6	Present	Present	Present	Absent
7	Present	Present	Present	Absent
10	Present	Present	Present	Absent
11	Present	Present	Present	Absent
12	Present	Absent	Present	Absent
13	Present	Absent	Present	Absent
14	Present	Present	Absent	Present
15	Absent	Present	Present	Absent

Clinical treatment consisted of the administration of perispinal injections of ozonized PRP into the longissimus dorsi muscle in both antimeres using a gauge needle with a 12-mm gauge and 4 cm long. In each antimer, ozonized PRP was administered at four equidistant points 10 cm from each other. Autologous PRP was obtained according to the technique described by Fantini et al. (2016). Ozone was generated using an ozone generator (Ozone & Life, São Paulo, Brazil), and a 10-mL O_2_O_3_ gas mixture (50 μg/mL O_3_ concentration) was collected in a 20-mL disposable syringe prefilled with the same amount of PRP. Intensive shaking was performed for 45 s. In all animals, three applications were performed at 15-day intervals.

Therapeutic ultrasound was applied using a standard ultrasound transmission gel for 20 min via a sound head with an effective radiating area of 10 cm^2^. The area received continuous ultrasound at 1 MHz and 1.0 W/cm^2^ according to the methodology described by Adair & Levine (2019).

Dynamic mobilization exercises were performed daily for the first week and in the following weeks on alternate days for two months. Dynamic mobilization consisted of three head movements with longitudinal cervical flexion (head on chest, head between the carpus and head between hooves), one head movement with cervical extension, and three head movements with lateral cervical flexion (head on shoulder, patella, and hock) to the right and left sides of the animal’s body, according to the methodology described by Oliveira et al. (2020). After assuming the correct posture for each exercise, the horses remained in this position for five seconds, with a snack (carrot) or grass leaves provided as a stimulus. These dynamic mobilization exercises corresponded to one series; each series was repeated five times per animal, adopting a 30-s rest interval between them.

Corrective shoeing was performed on all animals to restore foot balance. The procedure was performed at 30-day intervals by the same farrier under veterinary supervision.

Thirty days after treatment initiation, the animals gradually returned to physical activity using the following protocol: a) 5 min assembled by step in the first week; b) 10 minutes of assembled walking in the second week, followed by 5 min trotting; and c) 10 min step-by-step in the third week, 10 min trotting in a row, and 5 min walking. From the fourth week, a gradual return to the previous intensity of activity was recommended.

The treated animals were evaluated clinically and ultrasonographically at 15, 30, and 60 d after the start of treatment. In the assessment, it was determined that the horse presented relief of clinical signs of low back pain if the following three criteria were met: (a) if the clinical and ultrasonographic examination revealed regression of clinical signs associated with back pain, (b) if the horse was able to carry out its intended role normally, and (c) whether the rider thought the horse’s performance was acceptable or normal after treatment. The treatment was considered inefficient when at least one of the two people (veterinarian and rider) who carried out the evaluation thought that the horse did not show sufficient clinical improvement to meet the classification criteria for the improvement of clinical signs of back pain and performance.

All treated animals returned to sports activities at a higher level of performance than at the beginning of the treatment. Six months after treatment, telephone contact was made with the owner or trainer to determine if the back pain had relapsed. None of the animals relapsed during this period, and they could participate in vaquejada normally.

## Discussion

The objectives of clinical examination of the equine back are to determine whether back pain is present, the site or sites of pain, and the potential lesions responsible for the pain (Melo & Ferreira, 2020). Although back pain is a common cause of poor performance in equine in all disciplines, a standardized diagnostic workflow and a consolidated therapeutic approach are currently lacking (Riccio et al., 2018). The importance of this anatomic region in equine locomotion justifies investigations in this area, mainly those directed at therapeutic innovations (Fonseca et al., 2006).

In the equine dorsal region, there are two important epaxial muscles: the multifidus and the longissimus dorsi (LD). Equine with muscular disorders in the dorsal region show spasms in the LD during physical examination and work (Oliveira et al., 2015). All animals in the present report had some degree of pain on palpation of the thoracolumbar region and muscle hypertonicity, suggesting damage to the LD. On ultrasound examination, 14 animals were found to have LD myositis. Of the 15 horses evaluated, 13 had atrophy of the multifidus muscle, a common finding in equine back pain (Melo & Ferreira, 2020).

Identification and interpretation of clinical and ultrasound findings of injury to the epaxial musculature are of fundamental importance for the establishment of an adequate therapeutic protocol. In this series of cases, intralesional administration of ozonized PRP and therapeutic ultrasound were performed for the resolution of LD myositis and dynamic mobilization exercises were performed for multifidus muscle hypertrophy.

Therapeutic ultrasound showed an immediate effect in reducing or eliminating muscle spasms and pain on palpation of the thoracolumbar spine. According to Mikail & Pedro (2005), therapeutic ultrasound has thermal and nonthermal effects at the tissue level. In the first case, controlled heating produces analgesia, decreased joint stiffness, increased blood flow, increased collagen extensibility, and reduced muscle spasms. No thermal effects correspond to a mechanical action translated by pressure variations due to the passage of waves through the tissues, performing a micromassage.

PRP is a biological product obtained by centrifugation of autologous blood through a simple, cost-effective methodology to obtain high concentrations of growth factors with regenerative properties and, therefore, presents minimal risk of cross-contamination, disease transmission, or triggering immune reactions. In equine species, PRP has been studied in various diseases such as tendinitis, osteoarthritis, and skin wounds, with positive effects both individually and synergistically, improving cell migration and proliferation, angiogenesis, and matrix deposition in tendons and wound healing (Vendruscolo et al., 2012; Figueiredo et al., 2016; Pires et al., 2021).

Fifteen days after the first application of ozonized PRP, a considerable improvement in the ultrasound aspects of the epaxial musculature was observed, with a return to the normal pattern of echogenicity of the musculature. After 30 d of treatment, no change in echogenicity was observed. Additionally, pain on palpation of the spine and/or muscle hypertonicity were not identified (Table 2).

The PRP and ozone combination was shown to be effective in the treatment of LD lesions in this case series. This could be because the anti-inflammatory effects of ozone are supported by the release of anti-inflammatory growth factors from platelets in PRP (Yeprem et al., 2018).

The results observed in the present case series corroborate the findings of both experimental and clinical studies that demonstrate the effects of intralesional injection of PRP on muscle injuries. Overall, these previous studies reported better muscle regeneration, increased neovascularization, and reduced fibrosis (Wright-Carpenter et al., 2004; Hammond et al., 2009; Gigante et al., 2012; Quarteiro et al., 2015).

The rationale for the use of PRP is that additional growth factors released by platelets would augment the natural healing process (Hamid et al., 2014). In muscle cells, these growth factors promote the activation of satellite cells, increase the diameter of regenerative fibers, stimulate myogenesis, and increase the expression of myoD protein (38 kDa) and myogenin, in addition to accelerating the recovery time after strain injury (Vendruscolo et al., 2012).

It is believed that PRP is also capable of blocking the expression of nuclear factors and the action of pro-inflammatory molecules, such as interleukins, acting in an anti-inflammatory and analgesic manner (Yamada et al., 2012). These two actions may explain the absence of pain after 30 days of treatment and may have been enhanced by therapeutic ultrasound sessions performed on alternate days during the treatment period.

Quarteiro et al. (2015) highlighted that the concentrations of different growth factors in PRP obtained from different species (rats, rabbits, sheep, horses, and humans) show significant variation, which directly influences the results obtained in both experimental studies and clinical trials. Therefore, studies on standardized protocols are needed to increase knowledge about the effects of PRP used on the treatment of back pain in horses.

Ozone can be used to treat inflammatory and degenerative diseases of the musculoskeletal system, showing anti-inflammatory and antioxidant effects (Yeprem et al., 2018). This effect is achieved by modifying the decomposition of arachidonic acid in inflammatory prostaglandins. As a result, there is a decrease in inflammatory components and pain (Barbosa et al., 2020).

The objective of ozone therapy is to control acute, adequate, and transitional oxidative stress without causing chronic oxidative stress. Repeated small oxidative shocks activate and stimulate the antioxidant system, consequently creating resistance to oxidative stress by inducing the antioxidant system (Jaramillo et al., 2020).

The multifidus muscle is classified as a back-stabilizing muscle (Oliveira et al., 2014); therefore, it plays an important role in stabilizing the intervertebral joints, acting as a connection between the spine bones and avoiding micro movements related to the joint surfaces during locomotion. Atrophy of this muscle, as noted in this case series, can result in vertebral instability capable of promoting vertebral pathologies, such as osteoarthritis (Rodrigues et al., 2021). Therefore, it is imperative to develop therapeutic approaches that promote hypertrophy of this muscle.

Atrophy of the multifidus muscle in patients with back pain is common in both humans and horses (Melo & Ferreira, 2020), which corroborates the findings of this report. Atrophy is of the neurogenic type and selective for this muscle, not affecting other epaxial muscles. The asymmetry in the transverse area of the multifidus muscle between the right and left sides is a reliable indicator of back pain (Clayton, 2012).

Inhibition of multifidus activity promotes instability and micromovement of the intervertebral joints, predisposing to the development of other spinal disorders. To compensate for the loss of intervertebral stability, there is greater recruitment of the longissimus muscle, which often appears firm (tense) at palpation in horses with back pain, as reported by Melo and Ferreira (2020) and observed in this study.

In horses, the application of resistance exercise-based training programs is one of the main ways to promote muscle hypertrophy. These exercises are considered effective stimulators of muscle protein synthesis, stimulating the increase in the number of sarcomeres, as well as improving muscle fiber size and diameter. Muscle impairment associated with back pain is regarded as a motor control problem rather than a simple strength deficiency. Therapeutic exercises have been suggested to be based on a motor learning exercise protocol (Clayton, 2012).

The benefits of adopting a functional exercise-based physical training program have been extensively studied in recent years (Oliveira et al., 2015; Rodrigues et al., 2021) and have been shown to be effective in the therapeutic protocol in this series of cases. The observed benefits included increased balance, elasticity, and flexibility of the animals after 30 days of dynamic mobilization exercises, as described by Frick (2010). It is noteworthy, however, that all animals showed some degree of resistance at the beginning of the dynamic mobilization exercises. Dynamic mobilization exercises (DME) were performed for three months.

The DME protocol was effective in promoting multifidus muscle hypertrophy, as identified on the ultrasound evaluation of the lumbar musculature performed at 60 days. However, it should be noted that this assessment was made subjectively comparing the ultrasound images on the day of the initial clinical examination and after 60 days of treatment.

A consistent clinical finding in the equine in this study was the occurrence of hoof imbalances, especially in the hind limbs. In all animals, a mediolateral imbalance was observed, associated (90%) or not (10%) with negative plantar angle syndrome, which is consistent with the findings of Ridgway & Harman (1999), who reported that it is not uncommon for many sore-backed horses to have significant hoof imbalances. The occurrence of foot imbalances in horses involved in vaquejada has already been reported in the literature (Melo et al., 2011), and despite the advances that have taken place in relation to trimming and shoeing techniques, it still seems to be a frequent phenomenon predisposing horses to various musculoskeletal disorders (Melo et al., 2006; Dau et al., 2015).

The association between foot imbalances and the occurrence of equine back pain has been poorly studied. However, anecdotal reports from veterinarians and farriers suggest that this association is present.

The relationship between negative plantar angle syndrome and painful back conditions arises from the components of the posterior heel syndrome. A horse that has sore heels or chronic tendon or ligament strain in the posterior aspect of the leg assumes the posture of an “elephant standing on a ball,” with its front legs slightly pulled back to alleviate tension and discomfort. Even then, tension is held in the back muscles to help support the limbs. The back progressively loses its suppleness and flexibility, a situation that continues, leading to muscle strain and pain (Ridgway & Harman, 1999).

When the horse has a mediolateral imbalance, the load is on the medial aspect of the hoof and the lateral wall starts to flare outward (Kilmartin, 2015), corroborating the findings of this case series.

In this situation, the hindlimb abductors work to resist the tendency of the limb to adduct. The high medial wall and rotation of the hoof capsule contribute to the hoof twisting outward on propulsion. The hock twists, and the stifle rotates in. The hip joint is under load, and the increased torsion causes hip pain. Repeated work results in tension in the abductors and pain in the sacroiliac joint. Pain is also found in the lumbar spine. There is fascial tension over the paralumbar muscles, which extends down through the tensor fascia latae. The biceps femoris and deep gluteal muscles are under stress. The influence of the iliopsoas on the lumbar spine results in lumbar pain and restrictive vertebral units. This often extends to the transverse joints and reduces the effectiveness of the lumbosacral joint. It is not uncommon to find inflammation and edema when palpating the lumbosacral junction (Kilmartin, 2015). Stress on the biceps femoris and deep gluteal muscles can result in muscle pain identified on palpation, as noted in this report.

To rehabilitate the equine back, it is imperative that muscles be retrained and brought into a balanced state for riders to be correctly positioned on the horse (Ridgway & Harman, 1999). To accomplish this, therapeutic shoeing is utilized to rebalance the hoof. The type of horseshoe used for corrective shoeing varies depending on the type and degree of foot imbalance observed. Shoeing was performed for three months or until the imbalance was corrected with 30-d intervals between procedures.

The therapeutic protocol used in this series led to the clinical resolution of the disease, resulting in the return of the animals to competition. According to the evaluation carried out by the trainer or competitor, 100% of the horses returned to a level of performance superior to that observed before treatment. These results differ from those obtained by Melo and Ferreira (2020) when using a therapeutic protocol with perispinal corticoid injections.

No complications related to the adopted therapeutic protocol were observed during the follow-up period, which differs from the findings of Melo and Ferreira (2020) when using perispinal injections of triamcinolone to treat back pain in a series of 20 cases.

## Conclusions

The therapeutic protocol adopted was effective in promoting clinical regression of back pain, and no complications were observed. In addition, the protocol proved to be easy to perform and applicable to the field.
